# TGF-β-induced epithelial to mesenchymal transition drives proteoglycan binding specificity of HPV-based drug conjugates for cancer cells

**DOI:** 10.3389/fonc.2026.1918692

**Published:** 2026-09-21

**Authors:** Nathan R. Fons, Rhonda C. Kines, Parastoo Azadi, Stephanie A. Archer-Hartmann, Douglas R. Lowy, Elisabet de los Pinos, John T. Schiller

**Affiliations:** 1 Laboratory of Cellular Oncology, National Cancer Institute, National Institutes of Health, Bethesda, MD, United States; 2 Aura Biosciences, Boston, MA, United States; 3 Complex Carbohydrate Research Center, University of Georgia, Athens, GA, United States

**Keywords:** heparan sulfate proteoglycan (HSPG), human papillomavirus, tumor tropism, virus-like particle, virus-like drug conjugate

## Abstract

**Background:**

Human papillomaviruses (HPVs) utilize specifically modified proteoglycans (PGs) within the basement membrane as attachment factors prior to cell entry and infection. Similarly modified PGs are also found on cancer cells, which enables HPV with tumor-selective binding properties and highlights the potential development of HPV capsid-based anti-cancer therapies. In this study, we sought to investigate cancer-specific gene signatures and genetic factors that give rise to selective binding by HPV capsids.

**Methods:**

Using belzupacap sarotalocan (bel-sar; AU-011), an HPV virus-like drug conjugate (HPV-VDC) currently in clinical trials, we performed a cell binding screen using 115 cancer cell lines from which we performed gene expressions analysis. We developed drug resistant lines to investigate the developmental factors involved in tumor targeting.

**Results:**

We identified a strong association between drug binding and pathways involved in TGF-β-induced epithelial to mesenchymal transition (EMT). Furthermore, dose escalation treatments leading to selection of bel-sar resistant HeLa cells resulted in a marked reduction in TGF-β signaling and reversal of EMT, which was associated with a reduction in the synthesis and expression of heparan- and chondroitin sulfate proteoglycans.

**Conclusion:**

Overall, these data suggest that bel-sar binding is strongly dependent on the differential expression and modification of PGs that occurs during the EMT process. As many cancers undergo EMT during progression and invasion, these data provide mechanistic insights underlying the tumor-selective binding ability of HPV-VDCs. (200 words).

## 1 Introduction

Human papillomaviruses (HPVs) are a family of non-enveloped, double-stranded DNA viruses which infect actively dividing basal cells at sites of epithelial wounding (1). HPV particles initially bind secreted heparan sulfate- or chondroitin sulfate proteoglycans located within the exposed basement membrane, before transferring to host cell receptors for viral internalization and transduction (1–4). Interestingly, HPV pseudovirus (PsV) has been shown to selectively bind and infect a variety of cancer cell lines through direct interaction with cell surface proteoglycans (PGs), bypassing the requirement for initial basement membrane interaction (4, 5). As primary, non-cancer-derived, cell cultures are unable to bind HPV PsV, it was proposed that cancer cell lines in particular, express specifically modified PGs which mimic those found within the basement membrane and facilitate viral binding (5, 6). This observation has prompted the development of HPV capsid-based anti-cancer therapies such as belzupacap sarotalocan (bel-sar; AU-011)), which is currently in clinical trials for the treatment of choroidal melanoma and bladder cancer (7, 8).

Proteoglycans are cell surface-expressed or secreted glycoproteins which are comprised of a protein core covalently attached to at least one glycosaminoglycan (GAG) chain, such as heparan sulfate (HS), chondroitin sulfate (CS), keratan sulfate (KS), or dermatan sulfate (DS) (9). While all types of PGs are expressed in humans, heparan sulfate proteoglycans (HSPGs) and chondroitin sulfate proteoglycans (CSPGs) especially, make up a diverse family of receptor molecules that participate in many important cellular processes, including cell growth, signal transduction, angiogenesis, and adhesion (10–12). HS and CS chain biosynthesis involves a series of semi-overlapping enzymes that regulate initiation, elongation, and sulfation of the growing polysaccharide, which can sometimes reach chain lengths of up to 250 disaccharides (10, 13, 14). Both the length and sulfation pattern of the resulting GAG chain are crucial for the function of PGs, providing binding sites for growth factors (e.g. FGF, VEGF, TGFβ) as well as for extracellular matrix (ECM) components (e.g. collagen, integrins, and fibronectin) (9, 10, 12). Given their vital functions in integrating extracellular cues, it is unsurprising that alterations in the expression and modification of CSPGs and HSPGs have been identified as driving events in cancer development and progression (15, 16). One such driving event relates to the regulatory roles that PGs play during the process of epithelial to mesenchymal transition (EMT) (17). EMT encompasses a variety of global cellular events that occur as cells switch from epithelial to mesenchymal phenotypes, such as changes in gene expression, behavior, and overall morphology (18). During EMT, epithelial cells lose their characteristic tight cell junctions, apical-basal polarity, and overall adhesion to the basement membrane, developing instead a flatter, fibroblast-like morphology with increased migratory and invasive capacity (18, 19). EMT is an essential process for many normal-physiological conditions, including embryonic development, stem cell maintenance, and wound healing (20). However, EMT also occurs in pathogenic settings, such as cancer, where it promotes local metastasis and resistance to therapies (18–21). Both EMT and its reciprocal process, the mesenchymal to epithelial transition (MET), are reversible events, stimulated and enacted by a variety of extracellular cues and effector signaling molecules (18–20). These processes rarely result in complete phenotypic transitions, and instead manifest as a continuum of intermediate states, with cells possessing mixtures of both epithelial and mesenchymal characteristics (18, 22).

Many PGs, including members of the syndecan and glypican family of proteins, are notable participants in EMT and found to be upregulated during the transition from epithelial to mesenchymal phenotypes (17). Their functional role in focal adhesion formation and binding to ECM components can directly control cell morphology, cell adhesion, and migration, all key hallmarks of EMT (18, 23). In addition to these contributions, PGs also play a regulatory role throughout the EMT process, via interactions with stromal cytokines such as TGF-β (17). Perhaps the most well-known, and best studied, of the many EMT-inducing growth factors, TGF-β activates SMAD-dependent and SMAD-independent signaling pathways to drive the expression and repression of hundreds of genes (19, 24). Genes induced by TGF-β include important transcription factors SNAIL, SLUG, and TWIST1, which act to repress epithelial genes, such as E-cadherin (CDH1), and stimulate the expression of numerous mesenchymal genes, including N-Cadherin (CDH2), vimentin (VIM) and all three known TGF-β isoforms, ultimately driving EMT (19, 25). In addition to these classic EMT markers, TGF-β has also been found to regulate the expression of cell surface and secreted PGs, laying the framework for many of the phenotypic changes that occur during the EMT process (17). Interestingly, TGF-β activity can be controlled by PGs at a variety of levels. HSPGs act as crucial coreceptors for FGF, IGF-1, and Wnt receptor activation (26). These signaling pathways crosstalk with downstream SMAD and non-SMAD pathways and can modulate TGF-β activity in a context-specific manner (26). Extracellularly, PGs can control the activation of TGF-β from latency and regulate its overall bioavailability. For example, latent TGF-β relies on the presence of secreted PGs (e.g. perlecan, shed syndecans etc.) in the ECM for binding prior to activation by cellular proteases or integrin-mediated processes (27, 28). Additionally, stromal inhibitors such as biglycan (BGN) and decorin (DCN), two small leucine-rich proteoglycan (SLRP) Class I family members, tightly bind and sequester TGF-β, reducing its availability to activate neighboring cells (27, 29). Interestingly, the chondroitin and dermatan sulfate chains present on BGN and DCN, are known to interfere with this TGF-β binding ability. Therefore, in addition to the roles described for the different cell surface and secreted PGs, their associated GAG chains may also provide additional levels of control over TGF-β activity.

Despite widespread pre-clinical and clinical evidence for PGs as mediators of oncogenic signaling, less is known about the exact GAG modifications that occur during cancer development and how they contribute to the binding affinity of these pleiotropic receptors. Insights here could provide a better understanding of general tumor biology as well as afford detailed information regarding specific GAG binding motifs which can be therapeutically exploited by proteoglycan-targeted anti-cancer therapies. One such promising therapy, bel-sar, is an HPV-based virus-drug conjugate (VDC) comprised of an HPV-derived virus-like-particle conjugated to a photoactivatable infrared dye. Bel-sar has been shown to directly bind cancer cells via cell surface PG receptors and upon photoactivation, promote immediate reactive oxygen species (ROS)-induced necrotic cell death (7, 8). In this study, we wished to investigate the transcriptional signatures and PG/GAG biomarkers that are responsible for the cancer-selective binding properties of bel-sar. We found that enhanced drug binding is strongly associated with TGF-β-induced EMT expression signatures across a wide array of different cancer cell lines. Additionally, we observed that inhibition of TGF-β signaling is accompanied by a global loss in HS and CS expression and can result in resistance to HPV-VDC treatment *in vitro*. Overall, this work suggests that PGs and GAGs are dynamically and specifically modified throughout the EMT process, especially as a result of TGF-β signaling. As many cancers, especially metastatic tumors, undergo EMT, it is thus these EMT-associated PG/GAG modifications which ultimately drive the broad, yet tumor-selective, tropism of HPV-VDCs.

## 2 Methods

### 2.1 Cell culture and reagents

All culture conditions and sources of the cell lines for the binding screen are listed in **Supplementary Table 1**. HeLa cells and derived clones were also cultured in DMEM + 10% FBS and sub-passaged every 3-4 days to maintain sub-confluency. TGF-β1 (R&D biosystems cat: 7754-BH) was used at 10 ng/mL diluted in DMEM + 10% FBS, unless otherwise stated. SB431542 (Selleckchem cat. S1067) was used at a final concentration of 4 µM and also diluted in DMEM + 10% FBS. Bel-sar was acquired from Aura Biosciences. Heparinase I (HepI; cat. H2519), Heparinase III (HepIII; cat. H8891), and chondroitinase ABC (ChABC; cat. C3667), were all purchased from Millipore-Sigma and reconstituted according to the manufacturer’s recommendations (PBS + 50 mM NaCl + 4 mM CaCl_2_ + 0.01% BSA).

### 2.2 Bel-sar binding screen

Assessment of bel-sar binding capacity across the cell panel was performed as described previously (7). In brief, cell lines were harvested using 10mM EDTA, recovered for 2 hrs in culture media at 37 °C on a nutating rocker and seeded in 96-well round bottom plates at 2x10^6^ cells/mL. Cells were then washed with media, before adding a serial dilution range of bel-sar doses (200 µL each, starting at 25 µg/mL) made in PBS + 2% FBS. Cells were then incubated for 1 hr at 4 °C and washed two times with PBS + 2% FBS to removed unbound particles. Cells were resuspended in 200 µL of phenol-red free DMEM + 10% FBS and to test bel-sar cytotoxicity, 100 µl of each sample was transferred to a black 96-well plate for treatment with 25 J/cm^2^ near infrared light (NIR) (Modulight ML6700-PDT with MLA Kit). The remaining 100 µL of cells were kept in the dark and served as the baseline control. Cells were allowed to recover for 1 hr at 37 °C followed by Live-Dead Yellow staining (ThermoFisher Scientific cat: L34968) for 20 mins at RT, followed by a PBS + 2% wash and fixation with 4% PFA for 10 mins at RT. Cells were then washed two times with PBS + 2% FBS prior to detection on a FACS Canto II flow cytometer (BD). FlowJo Analysis software was used to analyze the geometric mean fluorescent intensity (GMFI) of the cell-bound bel-sar using the conjugated fluorescent dye and potency was determined using the Live-Dead stain. Using GraphPad Prism to plot these values, binding and potency dose response curves were generated. Area under the dose response curve (AUC) was then calculated using GraphPad prism to generate final binding and potency capacity AUC values from the bel-sar dose range between 0 µg/mL - 7.5 µg/mL.

### 2.3 Gene expression data and bioinformatic analysis of cell binding screen

Gene expression data for the cell panel was acquired from the Dependency Map Project (https://depmap.org/portal/) which contained RNA sequencing expression (RSEM) values and cell line metadata (originating tumor type, RRIDs, etc.) for all cell lines used in the analysis. Data was analyzed in R, using packages: ggplot2, GSVA, clusterProfiler, DESeq2, msigdb, and pheatmap, among others. Analysis code is available upon request. Gene sets used for GSEA were imported from the Molecular Signatures Database (MSigDb). GSEA was performed on a pre-ranked gene list, derived from the Spearman correlation coefficient of log_10_(bel-sar AUC) and variance stabilized transformation (VST) of gene expression RSEM values for each gene. Epithelial and Mesenchymal defining gene sets used for k-means clustering were acquired and used as described previously for determination of EMT intermediate states (22, 30). Gene-gene correlation studies were performed comparing the Pearson correlation coefficient of gene expression for canonical EMT markers and PG/GAG genes (**Supplementary Table 2**). Hierarchical clustering was then performed and plotted in R to subgroup the genes based on expression similarities.

### 2.4 Bel-sar resistance generation

To create Bel-sar resistant HeLa clones, parental HeLas were split into 4 unique cultures (RxHeLa, RxA, RxB, and RxC) of which RxA, RxB, and RxC were treated 14 times with increasing bel-sar doses (from 0.175 – 15 µg/mL). Treatments were spaced on average 7 days apart to allow time for cell regrowth, with cells split in between treatments as needed. Bel-sar treatment consisted of harvesting each clone with Accutase (Innovative Cell Technologies Inc. cat. AT104) and aliquoting 3x10^6^ cells into 15 mL centrifuge tubes. Cells were then washed with DMEM + 10% FBS and spun to remove supernatant, followed by treatment with the pre-determined bel-sar dose diluted in 3 mL DMEM + 10% FBS. Next, the cells were incubated at 4 °C for 1 hr with intermittent mixing, spun to remove unbound drug, and washed three times with phenol-red free DMEM + 10% FBS. The cells were then resuspended in 3 mL phenol-red free DMEM + 10% FBS and transferred to a 35 mm plate, which was treated with 25 J/cm^2^ NIR. Treated cell solutions were then placed in new 15 mL tubes, with 3 mL of fresh culture media, and allowed to recover for 1 hr, shaking at 37 °C, prior to replating in new culture dishes for resistance outgrowth. To note, RxHeLas were co-passaged for the duration of the study but were not treated. On even-numbered treatments, 200 µL of the recovered cells were seeded into 96-well plates for bel-sar binding and Live-Dead viability assessment as described above. Additional viability measurements were made at the conclusion of the resistance treatments using CellTiter-Glo 2.0 (Promega cat. G9242) according to manufacturer’s recommendations. After treatments 1, 5, 10, and 14, cell pellets were collected from treated cell plates, and RNA was extracted using an RNeasy mini kit (Qiagen cat. 74104). RNA sequencing was run using a NextSeq 550 instrument on poly A/mRNA enriched samples, and gene expression was analyzed from raw read counts quantified via mapping transcripts to human GENCODE v33 using salmon v1.1.0. Gene expression analysis was performed using R to calculate GSEA and GSVA enrichment scores, as well as generate plots. Analysis scripts are available upon request.

### 2.5 Western blotting

To assess protein expression of canonical EMT markers, cells were harvested using 10 mM EDTA, washed with PBS, and spun to form pellets. Cell pellets were then lysed with RIPA buffer (150 mM NaCl. 1% NP40, 0.5% sodium deoxycholate, 0.1% SDS, 50 mM Tris base, 1 mM EDTA) for 25 min on ice, followed by spin clarification and protein quantification using Pierce BCA protein assay kit (ThermoFisher Scientific cat. 23227). Lysates were then diluted in RIPA buffer, NuPAGE sample reducing agent and LDS Sample buffers (ThermoFisher Scientific cats. NP0009, NP0007), prior to loading on a NuPAGE 4-12% polyacrylamide gel (ThermoFisher Scientific cat. NP0322). Gels were transferred to PVDF membranes (Millipore-Sigma IPVH85R) and then blocked at RT for 1 hr in 5% BSA (Millipore-Sigma 2980-1 kg). Next, blots were incubated with primary antibodies diluted 1:1000 in 5% BSA, unless otherwise indicated, and rotated overnight (O/N) at 4 °C. Antibodies were acquired from Cell Signaling Technology (E-Cadherin #3195; VIM # 5741; SNAIL #3879), Santa Cruz Biotechnology (N-Cadherin sc-59987), and ProteinTech Group (GAPDH-HRP cat. hrp-60004 *diluted 1:5000 in 5% milk). After O/N incubation, the blots were washed 3 times with Tris buffered saline + 0.1% Tween 20 (TBST), and incubated with secondary anti-rabbit or anti-mouse IgG antibodies (Cell Signaling Technology #7074 and #7076, respectively) diluted 1:3000 in 5% BSA for 1 hr. Blots were then washed three times with TBST, and protein bands were detected using Western Lightening ECL detection reagent (Millipore-Sigma NEL105001) on an Amersham Imager 600 (GE). Western blots for the detection of SMAD2 phosphorylation were performed as described above, from cell pellets harvested after 1 hr treatment with 10 ng/mL TGF-β1 using 0.05% trypsin as the detachment reagent. Phospho-SMAD2 and total SMAD2 antibodies were purchased from Cell Signaling Technology (cats. #3108 and #3102, respectively).

Detection of secreted biglycan and decorin was performed using concentrated conditioned media from the indicated cell cultures. Cells were seeded at 1x10^6^ cells in T75 flasks and grown for 72 hrs prior to conditioned media collection. The collected medias were then spun at 2000 xg for 5 mins to remove any floating cells and transferred to Amicon Ultra-4 30 kDa MW cut-off filters for concentration according to the manufacture’s recommendation. The concentrated supernatants were collected and diluted in PBS to a final volume equivalent to 50-fold concentration. Samples were than diluted in reducing agent and LDS sample buffers for western blot detection as described above. Anti-BGN and DCN antibodies (R&D Biosystems cats. AF2667 and AF143 respectively) were diluted 1:1000 while HRP-conjugated, donkey anti-goat IgG (ThermoFisher Scientific cat. A15999) was diluted 1:10,000, both in 5% BSA.

### 2.6 SMAD binding element luciferase reporter and TGF-β signaling activity

The SBE4-Luc plasmid, (a gift from Bert Vogelstein Addgene plasmid #16495) (31), was co-transfected into our HeLa-derived lines using Lipofectamine 2000 (ThermoFisher Scientific cat. 11668019), with a control pNL1.1.TK[Nluc/TK] (Promega) reporter plasmid at a 100 ng/10 ng ratio. Transfected cells were incubated for 16 hrs at 37 °C, after which the media was replaced. Cells were then treated with 10 ng/mL TGF-β1 and incubated for 24 hrs at 37 °C, before lysis and luciferase detection via the Nano-Glo Dual-luciferase Reporter Assay system (Promega cat. N1521). Luciferase signal was detected using a Synergy H1 plate reader (Biotek) and normalized relative light units (RLUs) were calculated according to the manufacturer’s guidelines, dividing RLU from the SBE4-Luc reporter by the pNL1.1.TK nano-luc signal for each well.

To assess the transcriptional effects of TGF-β1 treatment within our isogenic HeLa models, cells were treated with 10 ng/mL TGF-β1 or 4 µM SB431542 for 72 hrs, prior to cell harvest and pelleting. RNA was extracted and sequenced as described above, using a NextSeq2000 instrument. Gene expression differences and analysis was performed in R and plotted using ggplot2 and GraphPad prism.

### 2.7 GAG isolation

Whole cells were lysed by physical disruption and brief sonication (30 sec on, 60 sec off for 5 mins on ice). The samples were then suspended in 2 mL 0.1 M Tris-HCl, pH 8.0, containing 2 mM CaCl_2_ and 1% Triton X-100. To this, pronase (Roche) was added to bring the final concentration up to 0.8 mg/mL, and the tissue was digested with shaking at 50 °C. After 24 hrs a second, 1.6 mg, aliquot of pronase was added and digestion continued for another 24 hrs. Finally, the enzyme was inactivated by heating to 100 °C for 15 mins. The buffer was adjusted to 2 mM MgCl_2_, benzonase (100 mU) was added, and the sample was incubated for 2 hrs at 37 °C. After inactivation of the enzyme (15 mins, 100 °C) any undigested material was removed by centrifugation for 1 hr at 4000 xg.

The supernatant containing whole free GAGs was applied to a DEAE-Sepharose –column (Cytiva, 2 mL free bed volume), washed with ~10 column volumes of loading buffer (20 mL, pH 8 Tris Buffer, 0.1 M NaCl). The sample was passed through the column, reapplied, and the bound GAG was washed with 10CV (20 mL) loading buffer. The sample was then eluted in 3 column volumes of elution buffer (6 mL, pH 8 Tris Buffer, 2 M NaCl). The eluate was collected and lyophilized to dryness. Remaining salts were removed with a commercial PD-10 column (Cytiva), and then re lyophilized before disaccharide analysis.

### 2.8 Disaccharide analysis and GAG quantification

An aliquot of purified GAG material was selectively digested with either a blend of Bacteroides Heparinases I-III (specific for Heparan/Heparin Sulfate; New England Biolabs), Chondroitinase AC from Flavobacterium heparinum (specific for Chondroitin Sulfate; Sigma), or Chondroitinase B from Flavobacterium heparinum (specific for Dermatan Sulfate; Sigma) in a pH 7.0 Tris-HCl and incubated for 24 hrs at 37 °C. The enzyme was heat-inactived at 100 °C for 5 mins and then centrifuged at 14,000 rpm for 30 mins to pellet any protein before introduction to the HPLC.

The enzymatic products were then separated with SAX-HPLC (4.6 × 250 mm Waters Spherisorb analytical column with 5 μm particle at 1.0 ml/min flow rate) coupled to fluorescence detection via post-column derivatization using methods previously described (32). The separations of sample disaccharides were compared to the separation of standard disaccharides (Galen Laboratories) for identification and quantification.

Cell surface HS and CS was also measured by cell staining with anti-HS 10E4 (Amsbio cat: 370255-1) and anti-CS-56 antibodies (Millipore-Sigma cat: SAB4200696). Cells were harvested with 10 mM EDTA, washed once with DMEM + 10% FBS, blocked with PBS + 10% FBS, and incubated for 1 hr at 4 °C with either antibody, diluted 1:200 in culture media. Next, cells were washed three times with blocking buffer and incubated with secondary anti-mouse IgM-488 antibody (Invitrogen cat: A21042) for 1 hr at 4 °C. Cells were then washed again and fixed with 4% PFA prior to detection on a flow cytometer.

### 2.9 Cell imaging and scratch migration assay

To image morphologic changes across the HeLa clones, 2000 cells were seeded at per well in a 96-well plate, and incubated for 4 hrs at 37 °C. The cells were then left untreated, or treated with TGF-β1 (10 ng/mL) or SB431542 (4 µM) for 72 hrs at 37 °C. Following the treatments, cells were then washed with PBS prior to fixing with 4% PFA for 20 mins at RT. Next, cells were permeabilized with 0.1% Triton X-100 for 15 mins at RT and stained for 30 mins with HCS CellMask Green stain and Hoechst 33342 (ThermoFisher Scientific cats. H32721 and H3570, respectively), followed by imaging using a 10x objective and a CellVoyager CV8000 high-content microscope (Yokogawa).

For the scratch migration assay, HeLa-derived cell lines were seeded at 4000 (RxHeLa) or 6000 (bel-sar resistant clones) cells per well in a 96-well plate and incubated 72 hours until confluent. Scratches were then made across each well, using a p200 pipet tip. Wells were then washed with warm culture media, and incubated for 30 mins at 37 °C. Following this incubation, the plate was transferred to a Cytation7 automated imaging microscope (Biotek), which was kept at 37 °C and 5% CO_2_ throughout the duration of the experiment. Brightfield images of each well were acquired using a 4x objective every 30 mins for 24 hours. Images were then processed using Gen5 software, and total wound area was calculated for every well and time point. In addition, average velocity measurements were determined for each well, calculated as µm^2^ per second, for the first 12 hrs of imaging, corresponding to overall cell migration across the wound.

### 2.10 Binding assessments of bel-sar resistant clones

Measurement of bel-sar binding capacity for the HeLa-derived cell clones was performed as described above (see section entitled *Bel-sar binding screen)*, with the notable exception of bel-sar dilutions occurring in DMEM + 10% FBS. For assessment of bel-sar binding in high serum conditions, bel-sar was first pre-diluted to a final concentration of 2 µg/mL in DMEM containing varying concentrations of FBS, and incubated at RT for 30 mins prior to cell binding assessment. For glycosidase treatments, cells were seeded in a 96-well plate at 50 k/well and pre-treated with the different enzymes diluted in DMEM without FBS to a final concentration of 1 U/mL (HepIII) or 2 U/mL (HepI and ChABC). Enzyme treatments were performed at 37 °C for 1.5 hrs, after which bel-sar was added at a final concentration of 2 µg/mL and binding was assessed via flow cytometry as described above. When indicated, AUC was calculated as area under the GMFI intensity-dose curve, for bel-sar doses 0-10 µg/mL.

### 2.11 Data analysis, graphing, and statistics

Data was analyzed and plotted using R (gene expression correlation, volcano plots, hierarchical clustering, heatmaps, and spot density plots) or GraphPad prism (select bar/line plots, and non-linear regression fitting). Unless otherwise stated, data points represent the mean of three independent biological replicates, with error bars the standard deviation of the mean. Statistical significance was determined through Student’s t test performed using R or GraphPad prism. P-values are indicated in the figure legends.

## 3 Results

### 3.1 HPV-VDC binding correlates with EMT gene expression signatures

To investigate the broad tumor tropism of HPV-VDCs, we first performed a binding screen of 115 cancer cell lines, originating from 20 different tumor types, with the drug, AU-011 (bel-sar). Bel-sar binding was assessed via direct fluorescent detection of the conjugated dye on cells and quantified as a summed area under the dose range-intensity curve (AUC) (**Supplementary Table 1**). Overall, bel-sar displayed a remarkably broad binding affinity, strongly interacting to cell line representatives from nearly all tumor types (**Figure 1A**). Both central nervous system (CNS) and melanoma (labelled as skin) cell lines were strongly represented in the top binding quintile of the panel (**Supplementary Figure 1A**). As expected, hematopoietic cancers (lymphoid and myeloid), which are known to express very low levels of cell surface PGs (33), were almost completely devoid of binding and heavily enriched in the lowest binding quintile. Of note, the binding capacity of cell lines for bel-sar was strongly predicative of drug-sensitivity (pearson r^2^ = 0.70), supporting previous work that found cell surface binding is an essential pre-requisite for drug-induced cell killing (7) (**Supplementary Figure 1B**).

**Figure 1:**
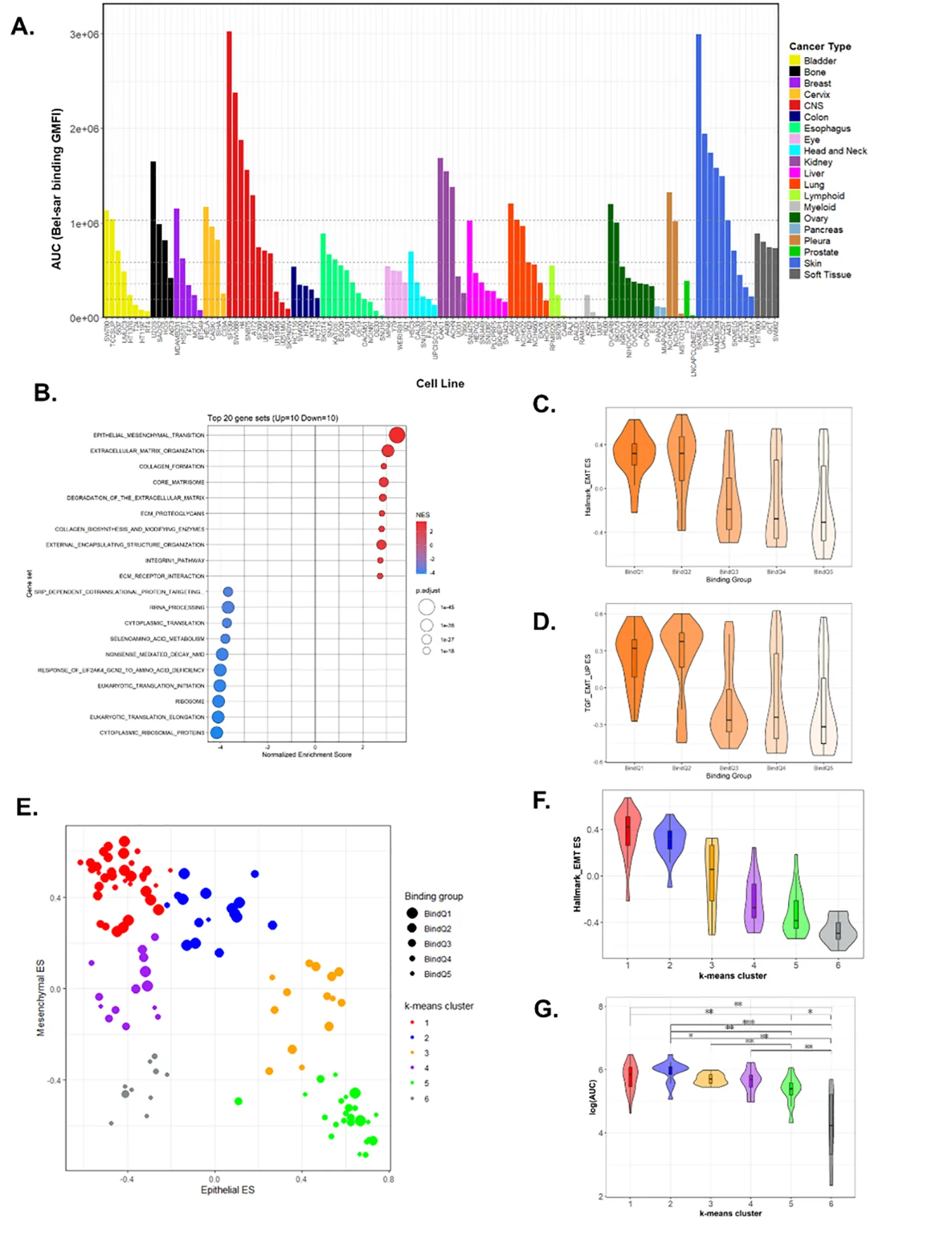
HPV-VDC binding correlates with gene expression signatures involved in the Epithelial to Mesenchymal Transition. **(A)** Bel-sar binding capacity of 115 cancer cell lines as measured by calculating area under the dose response curve (AUC) for bel-sar binding geometric mean fluorescence intensity (GMFI). Dashed lines indicate separations of binding quintiles. **(B)** Top ten positively and negatively enriched gene sets from Gene Set Enrichment Analysis (GSEA) using expression data acquired from the Dependency Map Project (34). **(C, D)** Gene set variant analysis (GSVA) enrichment scores (ES) of cells in A, sorted by binding quintile for Hallmark_EMT **(C)** and TGFB_UP **(D)** gene sets. **(E)** GSVA enrichment scores (ES) of epithelial- and mesenchymal-derived gene sets for cell lines in A, colored by k-means computed clusters. **(F, G)** Hallmark_EMT enrichment score **(F)** and Bel-sar binding of cell lines (log(AUC) **(G)**, *p < 0.05, **p< 0.01, ***p<0.001.

Next, we wished to explore the transcriptomic signatures and identify genetic biomarkers which underlie the susceptibility of cancer cell lines for HPV-VDC binding. RNA-seq expression data from the Cancer Dependency Map project (DepMap) (34) were available for all of the cancer cell lines tested and displayed a broad level of transcriptional diversity across the panel (**Supplementary Figure 1C**). Spearman correlation coefficients comparing binding capacity and gene expression were then used to create a ranked gene list for gene set enrichment analysis (GSEA) (**Supplementary Figure 2A**). Using more than 10,000 curated gene sets from the Molecular Signatures Database (35, 36), we identified the HALLMARK_EPITHELIAL_TO_MESENCHYMAL_TRANSITION gene set (henceforth labeled, Hallmark_EMT) as the most positively enriched gene set associated with bel-sar binding (**Figure 1B**; **Supplementary Figure 2B**). Interestingly, a number of ECM-related gene sets were also positively enriched, indicating a preference for cells with large-scale dysregulation of their extracellular environment. To assess how the Hallmark_EMT gene set was presented throughout the cell line panel, an enrichment score was calculated via gene set variant analysis (GSVA) (37) for all 115 cell lines. We found a strong positive association of Hallmark_EMT with bel-sar binding, with the top two binding quintiles displaying very high average enrichment scores (**Figure 1C**). Additionally, using previously described (24) and curated lists of genes over- or under-expressed during TGF-β induced EMT (TGF-β UP and TGF-β DOWN, respectively), we found a notable enrichment in TGF-β UP genes within our highest binding quintiles with little to no correlation associated with TGF-β DOWN genes (**Figure 1D**; **Supplementary Figure 2C**). Together, this suggests that EMT, and more specifically TGF-β-induced EMT, may drive genetic expression patterns that act to enhance the binding of bel-sar to cancer cell lines.

To understand how bel-sar binding capacity changes as cells progress through EMT, enrichment scores were generated as described previously (30) for epithelial-defining and mesenchymal-defining genes. These scores were calculated for all 115 cell lines, and k-means clustering was performed to subgroup each cell line into clusters corresponding to different intermediate stages of the EMT process (**Figure 1E**). To note, cell lines that displayed strong negative enrichment scores for both epithelial and mesenchymal gene lists, were classified as a non-EMT subgroup, which contained nearly all of the hematopoietic-derived cell lines. Expectedly, the k-means derived clusters comprised the full spectrum of the EMT process, as determined by their respective Hallmark_EMT enrichment scores (**Figure 1F**). Clusters 1 and 2 contained the most mesenchymal-like cells, followed by clusters 3 and 4, with cluster 5 containing the most epithelial-like cell lines. Bel-sar binding capacity was found to vary based on these defined EMT clusters and was significantly reduced in the more epithelial-like cell lines (clusters 3,4, and 5) compared to the mesenchymal-like cells (clusters 1 and 2), with the non-EMT cluster (cluster 6) having the lowest overall binding capacity (**Figure 1G**). Interestingly, the enrichment of TGF-β UP and TGF-β DOWN genes also varied drastically throughout these defined clusters and displayed important differences in TGF-β signaling between the intermediate EMT transitions states, as previously documented (30) (**Supplementary Figures 2D, E**). Increasing enrichment of TGF-β UP genes was associated with increasing mesenchymal enrichment scores, while increasing TGF-β DOWN was associated with reducing Epithelial enrichment scores. Altogether, these data demonstrate that bel-sar binding to cancer cell lines is strongly correlated with the transition through a TGF-β-induced EMT process, with enhanced binding occurring on cell lines with mesenchymal-like phenotypes.

### 3.2 HPV-VDC binding is driven by key PG/GAG genes that are differentially expressed during EMT

As the binding of HPV-VDC to cancer cells occurs through direct interaction with cell surface PGs and their associated GAG chains, we next sought to understand how the expression of PG/GAG-related genes is altered throughout the EMT process. To this end, we performed gene-gene correlation analysis followed by hierarchical clustering to find transcriptional associations between PG/GAG genes and known markers of TGF-β-induced EMT. We developed a curated list of 100 different PG/GAG genes involved in GAG biosynthesis, nucleotide activation and transport processes, as well as all cell surface expressed PG core proteins; for comparison with EMT markers (**Supplementary Table 2**). As predicated, the expression of TGF-β isoforms 1 and 2 were tightly linked with the canonical EMT markers CDH2, VIM, FN1, COL1A1, and TWIST1 (**Supplementary Figure 3A**; red triangles and asterisks, respectively). Surprisingly, there were 20 additional PG/GAG-related genes that fell within this EMT-defining clade, comprised mostly of HS/CS biosynthesis enzymes (such as EXT1, NDST1, CHSY3) and cell surface PGs (SDC2, NRP1, CD44). The expression of these genes correlated well with the EMT markers and TGF-β isoforms shown and indicated a functional relationship between TGF-β expression and the biosynthesis of HS- or CSPGs. Additionally, the expression of these HS/CS biosynthesis genes was found to change throughout EMT transitions states with notable enhancement in mesenchymal-like cell clusters (**Supplementary Figure 3B**). Overall, these results suggest that a subset of PG/GAG-related genes are dynamically regulated throughout the EMT process, and that it is these expression level changes which determine and promote bel-sar binding to mesenchymal-like cell lines.

### 3.3 Bel-sar resistant HeLa cells have inhibited TGF-β signaling

We next aimed to study the interplay of HPV-VDC binding factors in the context of a single isogenic cancer model, instead of an aggregate of numerous cancer types. Beginning with the EC_50_, an incremental step-wise bel-sar dose-escalation and selection approach was used to generate resistant clones. After 14 cycles, we successfully generated three unique HeLa cell clones (RxA, RxB, and RxC) which had markedly reduced binding compared to parental Hela cells (Par. HeLa) or a passage-matched control HeLa cell line (RxHeLa), and that were resistant to the cytotoxic effects of bel-sar treatment (**Figures 2A, B**). RNA was extracted at select time points throughout the dose-escalation treatments and sequenced to look at gene expression levels during resistance generation. Principal component analysis of the resulting data displayed a strong shift between the early treatment groups (untreated and treatment 1) and later treatments (treatments 5,10, and 14) corresponding with large transcriptional changes during the initial generation of bel-sar resistance (**Figure 2C**).

**Figure 2:**
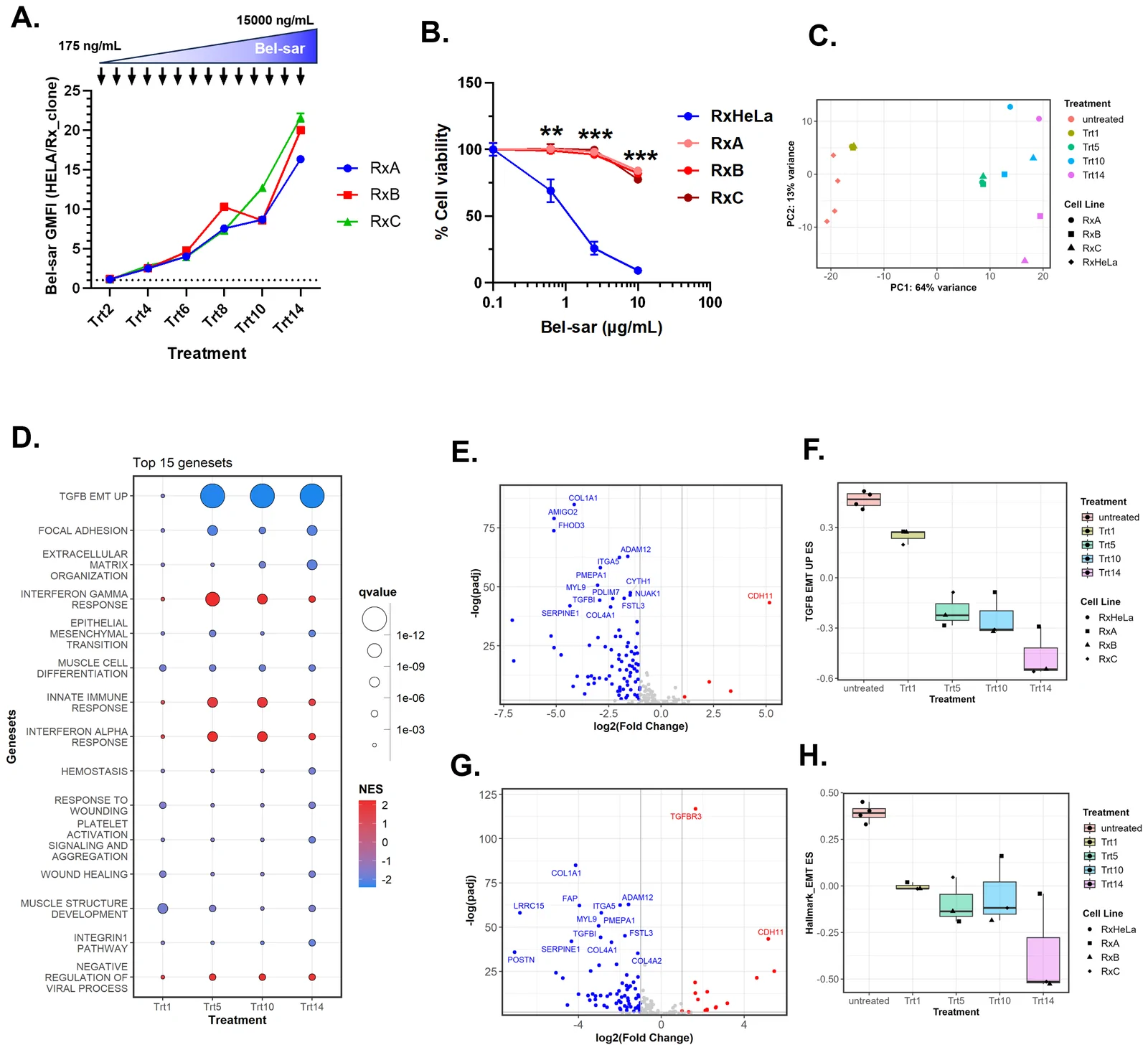
Bel-sar resistant HeLa cells have impaired TGF-β-induced EMT signaling. **(A)** Geometric mean fluorescence Intensity (GMFI) ratios of bel-sar binding in RxHeLa and bel-sar selected clones over duration of dose escalation treatments, treatments denoted with arrows. **(B)** Cell viability of bel-sar dose-escalated and selected clones and passage-matched RxHelas, 60 mins post bel-sar and infrared light treatment. **(C)** Principal component analysis of RNA-seq gene expression from bel-sar dose escalation treatments, comparing bel-sar selected clones and passage-matched RxHelas from indicated treatment doses. **(D)** Top 15 gene sets significantly enriched via GSEA across bel-sar selected clones from the indicated treatment number. **(E, G)** Differential gene expression of TGF-β UP **(E)** or Hallmark_EMT **(G)** gene sets comparing bel-sar selected clones and RxHeLa cells. **(F, H)** GSVA enrichment scores for the TGF-β UP **(F)** or Hallmark_EMT **(H)** gene sets across bel-sar selected HeLa cell treatments and RxHelas. **p<0.005, ***p<0.0001.

To identify dysregulated pathways that were shared among the resistance clones, we first performed differential gene expression (DGE) analysis, comparing the averaged gene expression levels of the HeLa clones with the passage-matched, but untreated control line, RxHeLa. From this, we utilized Ingenuity Pathway Analysis (IPA), to identify upstream regulators of the bel-sar resistance phenotype. Remarkably, inhibition of TGF-β1 signaling was predicted by IPA as the most significant upstream event driving the gene expression signatures seen in the resistant clones (**Supplementary Figures 4A, B**). Inhibition of SMAD3 and TGF-β3 signaling were also predicted as significant upstream events, supporting the regulatory role of TGF-β signaling on bel-sar binding. In addition to analysis using IPA, we performed GSEA from DGE-ranked gene lists for each of the different treatment groups. Using the previously mentioned 10690 gene sets, we also included the TGF-β UP gene set identified from our binding screen, to further ascertain the role of TGF-β in generation of bel-sar resistance. With this approach we found a significant negative enrichment of the TGF-β UP pathway (q < 1e-17 to q < 1e-21) and its associated genes in the resistant clones, starting after treatment 5 and continuing through treatment 14 (**Figures 2D, E**). This negative enrichment was further verified from GSVA-calculated TGF-β UP enrichment scores, where we observed a progressive reduction in EMT score for each of the three clones throughout the dose escalation treatments (**Figure 2F**). As expected, the Hallmark_EMT gene set was also negatively enriched in the resistant clones, albeit to a lesser degree, and displayed similar patterns of genetic repression (**Figures 2G, H**).

In the absence of TGF-β or other EMT-inducing signaling events, cells can often become more epithelial-like, through the process of MET (21). To assess the possibility of MET in our resistant lines, we first looked at canonical markers of epithelial (E-Cadherin) and mesenchymal (N-Cadherin, SNAIL, and VIM) phenotypes (**Figure 3A**; **Supplementary Figure 4C**). Both parental and RxHeLas were found to have high levels of N-Cadherin, SNAIL, and VIM, with little-to-no expression of E-Cadherin; representative of mesenchymal-like cells. However, the bel-sar resistant lines maintained lower levels of N-cadherin and SNAIL and regained substantial expression of E-Cadherin; representative of a more epithelial-like phenotype. To note, the expression of mesenchymal markers in these resistant lines were still present, indicating that only a partial MET had occurred. In addition to the described differences in EMT markers, we also observed morphological differences between these isogenic models (**Figure 3B**). As expected for mesenchymal-like cells, the RxHeLas grew as broad, flat cells, spreading out throughout the plate. Alternatively, the resistant lines were much taller and grew in clumps, rarely spreading out, characteristic of epithelial-like cells. Interestingly, these morphologic differences were ameliorated upon treatment of the resistant lines with TGF-β, and enhanced in parental HeLas upon treatment with SB431542, a TGF-β receptor inhibitor, supporting the relevance of TGF-β depletion on these structural changes. Lastly, as increased migratory ability is a defining feature of mesenchymal cells, we performed a scratch migration assay, testing the ability of these HeLa models to efficiently close an *in vitro* wound. While both parental and RxHeLa lines migrated quickly to close the scratch wound, the resistant lines were markedly attenuated, closing the initial wound at an average rate of 800 uM^2^/min compared to over 2000 uM^2^/min for the parental HeLas (**Figures 3C, D**). Importantly, this wound closure was not due to large differences in division rate, as seen in an XTT viability assay (**Supplementary Figure 4D**). Altogether, these results demonstrate that TGF-β depletion is a defining feature of bel-sar resistance generation in HeLa cells and drives a partial MET process, resulting in a more pronounced epithelial-like phenotype.

**Figure 3:**
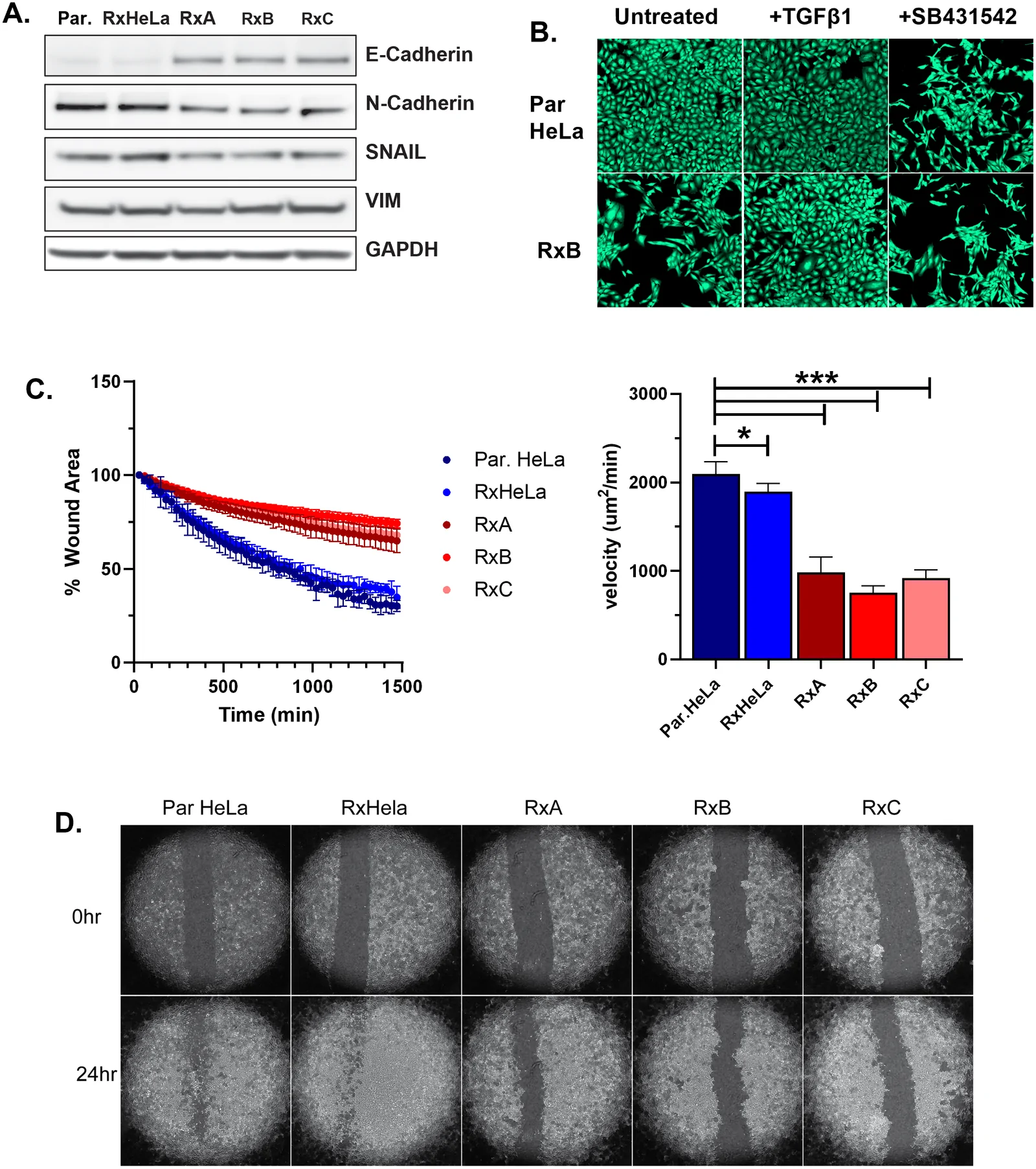
Bel-sar resistant HeLa cells have undergone a partial mesenchymal to epithelial transition. **(A)** Western blot of parental HeLas (Par.), RxHeLas, and bel-sar selected clones for EMT marker genes. **(B)** Representative images of parental HeLas and RxB cells untreated, or treated with TGF-β1 or a TGF-β inhibitor (SB431542), and stained with whole cell HCS CellMask stain. **(C)** Quantification of scratch wound healing rate (left) and calculated migration velocity (right) for bel-sar selected cell lines. **(D)** Representative images from wound healing assay in C at 0 and 24 hr time points post-scratch. *p<0.05, ***p<0.0001.

### 3.4 Surface expression of HS and CS is reduced on bel-sar resistant cell clones

We next chose to examine the bel-sar resistant clones in more detail by specifically looking at the cell surface expression of HS and CS chains, the predominant binding partners of HPV-VDCs. Disaccharide analyses were performed using SAX-HPLC, as described previously (32), to quantify total amounts of different sulfated HS and CS disaccharides. From this analysis, we identified a substantial reduction of total HS and CS in the resistant clones compared to the parental HeLa controls (**Figures 4A, B**). These results were further validated through cell surface staining with HS and CS-specific antibodies, where we again found a consistent reduction of these GAGs present on the bel-sar resistant cell lines (**Supplementary Figures 5A–C**). Notably, while total levels of HS and CS were depleted, only minor changes were observed in the composition of different sulfated species, indicating that total GAG loss and not differential sulfation, is likely responsible for the observed bel-sar resistance (**Supplementary Figures 5D, E**).

**Figure 4:**
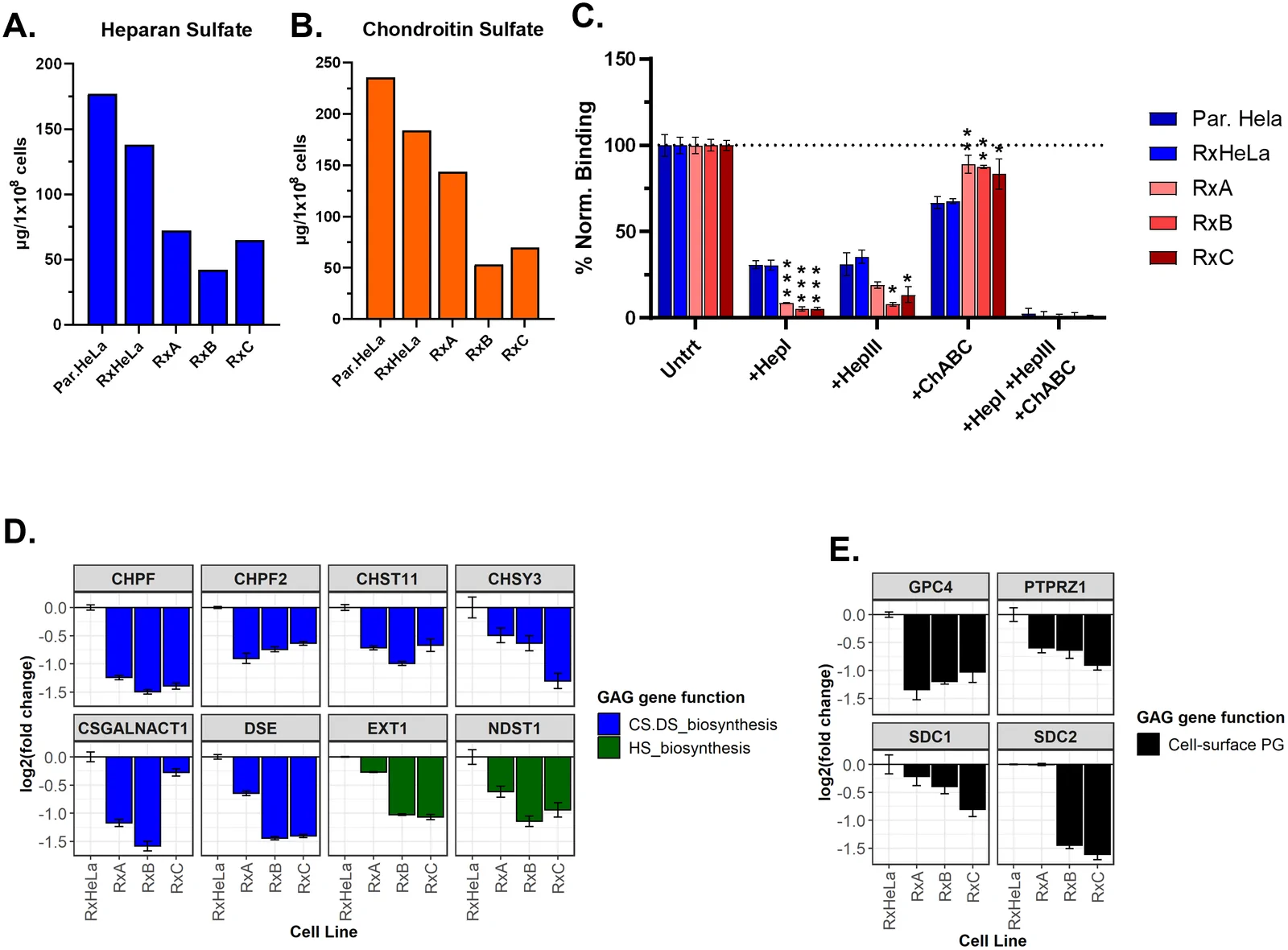
Bel-sar selected HeLa cells have reduced expression of HS/CS and cell surface proteoglycans. (**A, B)** HPLC-SAX analysis of cell lines, for total heparan sulfate **(A)** and chondroitin sulfate **(B)** disaccharides. **(C)** Normalized bel-sar binding of cell lines after treatment with heparinase I (HepI), heparinase III (HepIII), chondroitinase ABC (ChABC) or a combination of the three enzymes. **(D, E)** Gene expression of select GAG biosynthesis enzymes **(D)** and proteoglycan cores **(E)** comparing RxHeLa cells and bel-sar selected clones, RxA, RxB, and RxC. *p<0.05, **p<0.005, ***p<0.0001.

Considering these findings, we next wished to determine if the relative binding specificity of HPV-VDCs for HS or CS chains was altered in the bel-sar resistant clones. To ascertain binding dependencies, we used two different heparinases and a chondroitinase to enzymatically remove their cognate GAGs from the cell surface; and measured the effect on overall HPV-VDC binding. As expected, treatment of cells with heparinases (HepI and HepIII) and/or chondroitinase (ChABC) reduced bel-sar binding, with the greatest reduction seen in the heparinase-treated conditions (**Figure 4C**). Interestingly, bel-sar binding to the resistant cells was more sensitive to HepI and HepIII treatment, and less sensitive to treatment with ChABC, indicating a lower overall dependence on CS-receptors for their initial interactions with HPV-VDCs. This was further supported from binding experiments performed in high FBS conditions, where we found a near-complete loss of bel-sar binding to the resistant clones (**Supplementary Figure 5F**). As previously published, the binding of HPV virus-like particles to cells in high FBS conditions relies on surface expressed 6-O sulfated CS (4). Therefore, in addition to the global reduction of HS and CS expression, one notable feature of these bel-sar resistant models is a near-complete loss of the specific CSPG receptors used by HPV-VDCs for cell binding.

To understand why we observed such a pronounced drop in HS/CS levels in our resistant cell clones, we investigated possible transcriptional differences among enzymes within the HS and CS biosynthesis pathways. We found that while many genes were tightly controlled between our HeLa models, a select group of enzymes were downregulated in all three resistant clones. These genes included those involved in HS synthesis (EXT1 and NDST1) as well as CS synthesis (CHPF, CHPF2, CHST11, CHSY3, CSGALNACT1, and DSE) (**Figure 4D**). The transcription of select cell surface PG core proteins were also largely downregulated in some of the resistant lines, including SDC1, SDC2, GPC4, and PTPRZ1 (**Figure 4E**). These findings are consistent with the reduction in total HS/CS levels measured on the bel-sar resistant lines and suggest that transcriptional downregulation of these key biosynthetic genes can largely impair HPV-VDC binding and is a significant contributor towards the development of bel-sar resistance.

### 3.5 Repressed TGF-β signaling within bel-sar resistant clones results in transcriptional downregulation of select GAG biosynthesis enzymes

TGF-β has been known to induce the expression of a variety of proteoglycans and in some cases, GAG biosynthesis enzymes (17). Given the depletion of TGF-β signaling observed in the bel-sar resistant HeLa clones, we hypothesized that exogenous treatment with TGF-β may result in improved PG expression and ultimately rescue the binding of these cells to HPV-VDCs. Curiously, we found that while treatment of RxHeLas with a TGF-β inhibitor, reduced bel-sar binding by over 40%, the addition of TGF-β1 had very limited effects on the bel-sar binding of the resistant clones (**Figure 5A**; **Supplementary Figure 6A**). As outright disruption of TGF-β signaling could prevent these HeLa clones from responding to exogenous treatment with the growth factor, we decided to assess the overall functionality of TGF-β induced SMAD signaling in our model cells lines. We found that TGF-β was indeed functional in the resistant clones, as shown by the induction of SMAD2 phosphorylation and transcriptional activation of a SMAD binding element (SBE)-controlled luciferase reporter (**Figure 5B**; **Supplementary Figure 6B**). However, while TGF-β treatment induced comparable net changes in the activation of SBE transcription, reporter activity in the bel-sar resistant cell lines remained lower than similarly treated parental HeLas; characteristic of an upstream repression event.

**Figure 5:**
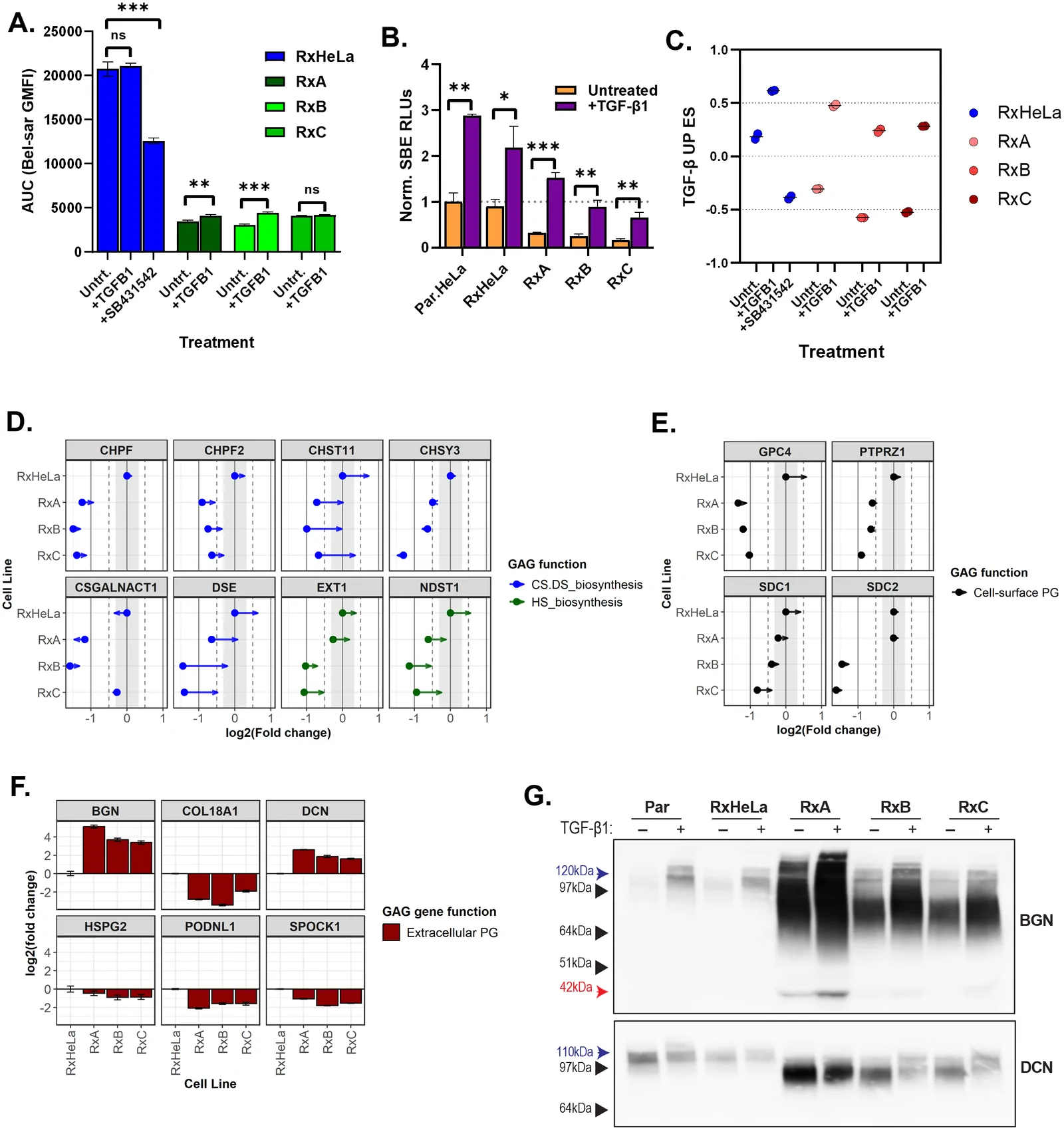
Impaired TGF-β signaling results in reduced PG/GAG gene expression. **(A)** Bel-sar binding in cell lines untreated or treated with 10 ng/mL TGF-β1 or a TGFβ inhibitor, SB431542. **(B)** SMAD-binding element (SBE)-reporter signal in parental (Par. HeLa), RxHeLa, and bel-sar selected HeLas (RxA, RxB, and RxC) untreated or treated with 10 ng/mL TGF-β1. **(C)** GSVA enrichment scores (ES) of TGFβ_UP gene set in cell lines treated as described in **(A, D, E)** Gene expression changes of select GAG-related genes **(D)** and cell surface proteoglycans **(E)** within bel-sar selected cell lines upon treatment with 10ng/mL TGF-β1, direction and magnitude of change denoted by arrow. Expression depicted as log2 of the fold change between cell clone and RxHeLa. **(F)** Expression of select extracellular proteoglycan genes comparing RxHeLa cells and bel-sar selected clones, RxA, RxB, and RxC. **(G)** Western blot of conditioned media from cell lines either untreated or treated with 10 ng/mL TGF-β1. Blue marker represents approximate fully glycosylated size, red marker indicates BGN unglycosylated size. *p<0.05, **p<0.005, ***p<0.0001.

To understand how TGF-β, and TGF-β repression, may be regulating the expression of cell surface PG/GAGs, we performed RNA-sequencing of our isogenic HeLa cell models, comparing untreated and TGF-β1 treated conditions. As expected, exogenous treatment with TGF-β1 resulted in consistent transcriptional shifts in both RxHeLa and bel-sar selected cell lines and increased TGF-β UP gene set enrichment scores; confirming that activation of TGF-β signaling was indeed functional in these cell lines (**Figure 5C**; **Supplementary Figure 6C**). Interestingly, TGF-β1 treatment had a wide-range of effects on the expression changes of PG/GAG genes in parental HeLa cells, with some experiencing large increases- or decreases in expression, and others experiencing no change (**Supplementary Figure 6D**). Looking specifically at the HS/CS synthesis enzymes previously found to be depleted in our resistant models, we again observed a diverse range of effects. While the expression of many previously depleted genes (CHPF2, CHST11, DSE, EXT1, and NDST1) increased in our resistant clones upon treatment with TGF-β1, others (CHPF, CHSY3, CSGALNACT1) did not (**Figure 5D**). This effect was also seen across PG core proteins depleted in the bel-sar resistant lines, with TGF-β1 rescuing SDC1 expression levels, but not SDC2, GPC4, or PTPRZ1 (**Figure 5E**). These findings highlight the complex nature of TGF-β induced expression changes of PG/GAG genes and suggest that bel-sar resistance in HeLa cells may be driven by two sets of genes, those induced by TGF-β signaling and those driven via TGF-β independent processes.

As TGF-β activity is regulated by a number of extracellular PGs, we next sought to examine the expression of secreted PGs by our resistant clones. We found that the transcription of many extracellular PGs was drastically altered in our resistant cells compared to the RxHeLa line, with substantial downregulation of COL18A1, SPOCK1, PODNL1, and HSPG2 (also known as perlecan), and upregulation of two Class I SLPR family members decorin (DCN) and biglycan (BGN), seen across our three resistant clones (**Figure 5F**). In line with previous observations, treatment with TGF-β was able to rescue much of these transcriptional differences, with the notable exception of biglycan (BGN) (**Supplementary Figure 6E**). Expression of BGN, a secreted CSPG, was found to be 10-30 fold higher in the resistant lines compared to RxHelas. Given the global repression of TGF-β present in these clones, this finding was surprising, as BGN expression is known to be strongly induced by TGF-β signaling. Indeed, TGF-β treatment of both parental and resistant HeLa cells resulted in greater BGN expression levels, and further exaggerated the differences between the resistant clones and untreated parental HeLas. Importantly, these transcriptional changes were validated at the protein level as well by testing conditioned media from our HeLa models either untreated or treated with TGF-β. As predicted from the transcriptomic data, the secretion of BGN, and to a lesser extent DCN, was found to be drastically higher in the resistant lines compared to the parental HeLa models, indicative of a large-scale upregulation of these two Class I SLRP family members (**Figure 5G**). Notably, the apparent molecular weight of the DCN and BGN western blot bands were also different between the parental and bel-sar resistant HeLa cells, with the resistant clones expressing proteins of smaller average molecular size. As the apparent gel-run sizes of DCN and BGN are largely dependent on total glycosylation level of these two proteins, such observations could suggest that DCN and BGN have lower total GAG content than the parental HeLas and support our previous work detailing a global reduction of HS/CS expression in these cells. Together, these findings contend that bel-sar resistance in HeLa cells is in part mediated by the over-expression and under-glycosylation of Class I SLRPS, including BGN and DCN, which inhibit TGF-β signaling, and reduce the expression of TGF-β stimulated PG/GAG genes.

## 4 Discussion

Overall, we have found that HPV-VDC binding to cancer cells is dependent on the cell surface expression of PGs that are upregulated during the process of EMT. Using an extensive panel of diverse cancer cell lines, we identified a strong association between TGF-β-induced EMT and binding with bel-sar. This connection was supported through examination of gene expression correlation approaches, which identified a subset of genes involved in HS and CS biosynthesis that are important for bel-sar binding and whose expression firmly correlated with EMT induced by TGF-β signaling. Additionally, we identified inhibition of TGF-β as a major driving event during the step-wise development of bel-sar resistance in HeLa cells. This loss corresponded with reduced levels of cell surface GAGs, a partial MET phenotype, and the downregulation of key PG/GAG-related genes. Lastly, we found that bel-sar resistance in our HeLa cells originated via both TGF-β dependent and independent processes. The concomitant loss of CS polymerization factors and the overexpression of Class I SLRPs, such as biglycan, work together to repress TGF-β signaling. This repression results in the dysregulation of many other HS and CS biosynthesis enzymes, further perpetuating the observed TGF-β inhibition, and driving overall resistance to bel-sar binding and killing (**Figure 6**).

**Figure 6:**
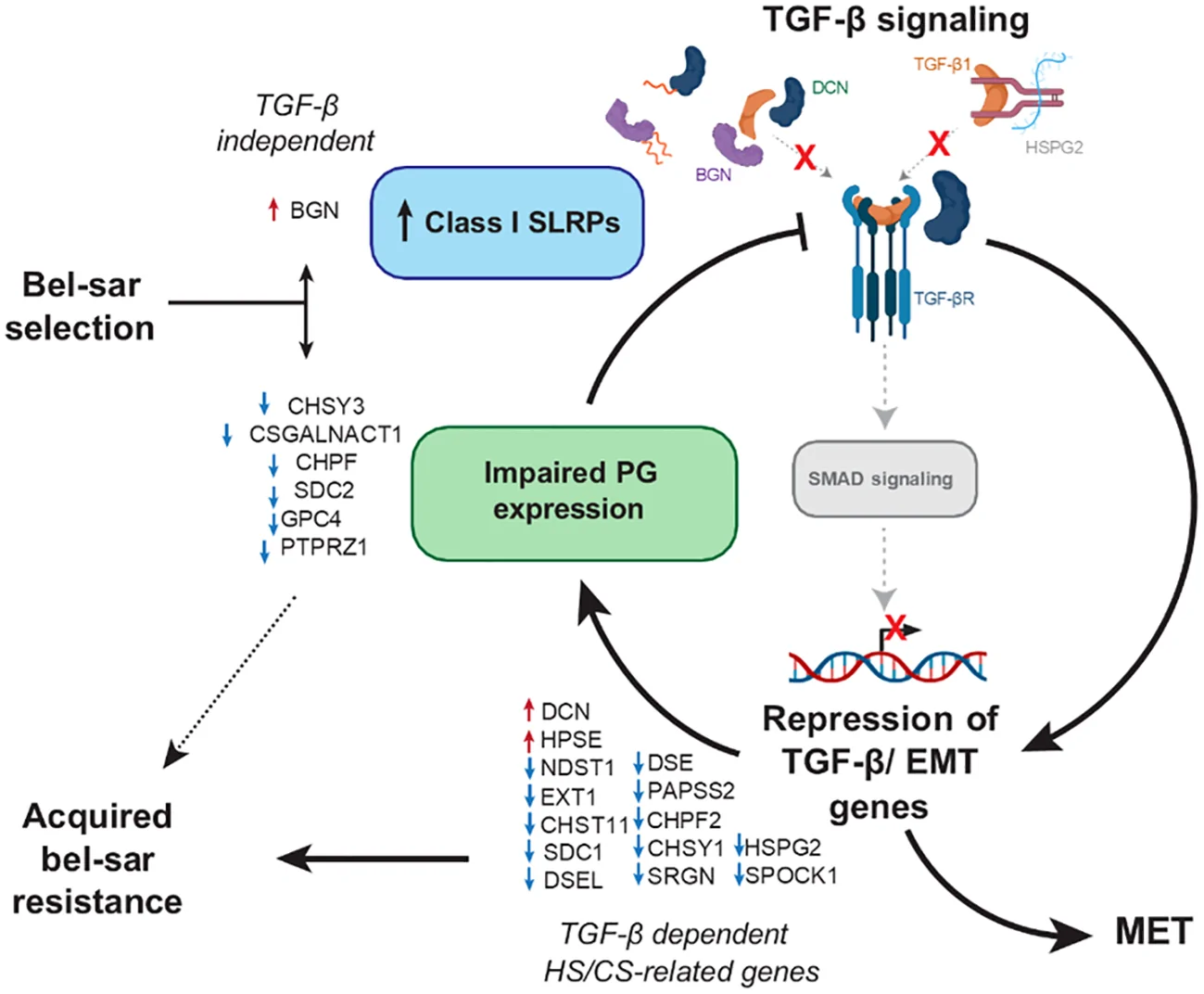
Bel-sar resistance in HeLa cells is driven by inhibition of TGF-β dependent and independent processes. Graphic model for the selection of bel-sar binding-impaired HeLa cell clones. Bel-sar dose escalation selects for cells which have reduced CS/DS expression, which decreases the overall glycosylation of Class I SLRPs, such as BGN, and enhances their TGF-β inhibitory abilities. The resulting inhibition of TGF-β signaling results in a partial mesenchymal to epithelial transition (MET), and a repression of numerous GAG biosynthesis and core proteoglycan genes. This reduction in PG/GAG genes further reduces the bel-sar binding capacity of HeLa cell clones and strengthens the inhibition of TGF-β signaling through the additional loss of glycosylation on SLRPs and HSPG2. Figure was generated using BioRender.

The binding affinity of bel-sar for PGs, or more specifically GAG chains, has been previously documented, and is derived from the main component of the VDC, an HPV virus-like-particle (8). Natural HPV infection occurs at sites of wounds, which expose both the basement membrane and basal epithelial cells to binding by the incoming virus (1, 8). In such wounding environments, the virus is surrounded by a multitude of host cells and factors which are tasked with repairing the epithelial barrier. One important aspect of this wound repair process is the induction of EMT in the surrounding epithelial cells. Platelets, invading immune cells, and adjacent epithelium all release cytokines, such as TGF-β, which induce EMT in the local epithelial cells, and promote cell migration and the overall re-epithelialization of the wound (38). Thus, it is likely that physiological HPV binding occurs in the presence of these EMT-inducing growth factors, and may in fact, have evolved to take advantage of them. In support of this, it has been well-documented that HPV interacts with a variety of extracellular enzymes and growth factors associated with a wounding environment, and that these interactions are required for proper viral entry and infection (39). Further, the influx of serum and platelet factors present in a wound may also determine the specificity of viral binding events. Previous work from our lab has shown that elevated serum or platelet factors effectively block canonical HSPG receptors on the cell surface and promote CSPG-mediated binding of HPV (4). While the potential advantages of CSPG-binding for viral infection remain to be elucidated, this change in receptor binding may result in altered tropism towards certain cell populations more suitable for viral proliferation. Interestingly, we found that all three bel-sar resistant HeLa clones completely lost the ability to bind the HPV-VDC in high serum conditions, and that enzymatic removal of CS chains from these cells had very limited impact on bel-sar binding compared to parental HeLas, even in normal media conditions. Altogether, this suggests that HPV-VDC interactions with cell surface CSPGs are likely important mediators of bel-sar induced cell killing and likely contribute to the enhanced binding observed on mesenchymal-like cancer cells.

As a dynamic regulator of EMT progression, TGF-β controls the expression of countless genes, including many well-characterized extracellular PGs (17, 24). Additionally, TGF-β has been shown to influence total GAG content of cells, indicating that underlying GAG biosynthesis may also be under its control (40, 41). There is mounting evidence that the levels of specific GAGs change during the EMT process, with the balance shifting from HS towards increasing CS content (42–44). Despite this, there remains much to be understood regarding the exact alterations that occur in GAG biosynthesis during EMT, as well as the genetic players involved. Here we show, that across a number of genetically diverse cancer cell lines, the expression of many GAG-related enzymes is strongly associated with transcriptional markers of EMT, including TGF-β1. Further, we show that TGF-β treatment in HeLa cells results in the enhancement of many of these GAG genes, including NDST1, EXT1, CHST11, and CHPF2, directly linking TGF-β signaling with the biosynthesis of HS and CS. As these GAGs perform a plethora of important cellular functions, the effects of TGF-β-induced changes in HS/CS expression expand well beyond HPV-VDC binding and likely influence the cellular binding and interactions with structural ECM proteins and cytokines. For example, FGF, Wnt, and IGF-1 all use HSPGs as co-receptors for activation and signaling through their canonical receptors (12, 26). This is of note, as these growth factors have been shown to modulate TGF-β signaling via crosstalk with downstream effectors and can act independently to initiate progression through EMT (20, 45). Thus, these HS-regulated growth factors may also have direct effects on the expression of GAGs and provide examples of how TGF-β independent processes may control the binding of bel-sar and other HPV-VDCs.

As mentioned previously, due in part to their pleiotropic growth factor binding abilities, GAGs and PGs have been associated as biomarkers for a number of different cancers (12). As a result of this link, targeting tumor-specific PGs is gaining momentum as a promising area in cancer therapeutics development (8, 46). With the notable exception of a monoclonal antibody that recognizes cancer-restricted CSPG modifications, identifying monoclonal antibodies that directly interact with the cancer-associated GAG chains has been challenging (47). This is due in part to the size and complexity of polysaccharides. GAG chains can extended up to 250 disaccharides in length and typically contain diverse stretches of sulfated and non-sulfated regions (10). It is thought that this linear spacing, as well as overall chain length and the individual array of sulfated sugars, are what determine binding affinity (10). Consequently, antibodies or other similarly-sized molecules, have binding epitopes that are generally too small to decipher these structural differences and are less effective at sampling the true range of cancer-targetable GAG modifications. HPV virus-like-particles, are large macromolecules, which contain 72 L1 pentamers, organized in an icosahedral capsid. Interaction with GAGs, namely heparan sulfate, has been shown to occur at multiple sites throughout the pentamer, with affinity growing as the HS chain length increases (48). Additionally, individual HS chains, or HS chains from multiple HSPGs are able to concurrently bind HPV capsids at different pentamers, which adds both binding strength to the overall GAG interaction (i.e. avidity), and allows for a diverse sampling of different modified GAG species (49). As a result of this, potentially small changes in overall GAG length or modifications can have sizeable impacts on the binding ability of HPV-VDCs for their PG receptor. Using SAX-HPLC, we measured a modest reduction (2-3 fold) of both HS and CS species in our bel-sar resistant HeLa models. Even when combined, these differences do not completely account for the approximately 15-fold difference in bel-sar binding observed in these cells and suggest that GAG chain length may also impact HPV-VDC interactions. This is further supported by the demonstrated drop in expression levels for genes such as EXT1, CHST11, CHPF, CHPF2, CHSY3, all of which play crucial roles in the polymerization of HS- or CS chains. Interestingly, the chain length of GAG polysaccharides may also dictate binding *accessibility*, as was described for rVAR2CSA, a recombinant malarial protein, which has shown strong cancer-specific binding properties (50). The binding of rVAR2CSA to oncofetal chondroitin sulfate was found only to occur on 4-O sulfated CS chains that were long enough to penetrate the cellular glycocalyx (51). Thus, the binding affinity of HPV-VDCs to cancer cells is likely a product of both quantity of cell surface PG receptors, as well as the length and accessibility of their associated GAG chains.

While the ability of bel-sar to bind cell surface PG/GAGs has been well documented, the underlying biology mediating its cancer-selective binding tropism has been to this point unknown. Here we identified progression through EMT as a major determinant of this tumor-targeting specificity. EMT plays a prominent role in cancer progression, resulting in mesenchymal-like cells with increased mobility and the capacity to invade surrounding tissue; ultimately supporting metastasis (18). Additionally, EMT has been shown to be important for cancer stem cell maintenance, promoting cell replication and resistance to many cancer therapies (21). Therefore, by specifically binding PGs that are expressed during EMT, HPV-VDCs, such as bel-sar, are able to target fundamental aspects of cancer biology. This provides an exceptional ability to bind and kill a wide variety of different tumor types, as well as reduce the chance of acquired clinical resistance. Such “pan-cancer” affinity was displayed during our cell binding screen, where we identified a large breadth of different solid-tumor types potentially targetable using this drug. This was especially true for CNS and melanoma-derived cell lines which displayed the highest bel-sar binding. As EMT plays a prominent role in early neural crest and melanocyte development, this result may highlight an important role for de-differentiation in PG biology, and ultimately, bel-sar binding (52). Cancer stem cells, which are pluripotent and are thought to be maintained in a de-differentiated state, also are known to undergo at least partial EMT and have altered expression of PG/GAG genes (53). Future studies should, therefore, examine the effectiveness of drugs, such as bel-sar, for selectively targeting and killing these notoriously treatment-resistant populations.

## Data Availability

The raw data supporting the conclusions of this article will be made available by the authors, without undue reservation.

## Author disclaimer

This research was supported [in part] by the Intramural Research Program of the National Institutes of Health (NIH). The contributions of the NIH author(s) are considered Works of the United States Government. The findings and conclusions presented in this paper are those of the author(s) and do not necessarily reflect the views of the NIH or the U.S. Department of Health and Human Services.
