# Supplementary material for: TGF-β-induced epithelial to mesenchymal transition drives proteoglycan binding specificity of HPV-based drug conjugates for cancer cells
Source: Front Oncol. 2026 Sep 21;16:1918692. doi: 10.3389/fonc.2026.1918692 (PMC13634947; doi:10.3389/fonc.2026.1918692)
Supplement: Supplementary file 1 [file DataSheet1.docx]

**Supplementary Figure 1. Bel-sar binds and kills a broad range of cell lines from different cancer types**. **A**. Bel-sar binding capacity of 115 cancer cell lines with binding quintiles denoted; Binding AUC: area under the bel-sar dose response curve. **B**. Comparison of bel-sar binding (logAUC) and bel-sar potency (Potency AUC) as measured by area under the dose response curves for each measure. **C**. Principal Component Analysis of the cancer cell panel in A; colored by cancer type and sized according to binding quintile.

**Supplementary Figure 2. Bel-sar binding correlates with gene expression signatures involved in TGF-β induced EMT**. **A**. Ranked list of genes ordered by the spearman correlation coefficient of gene expression and bel-sar binding. **B**. GSEA enrichment plot of the Hallmark_EMT gene set across ranked list in A. **C**. Gene set variant analysis (GSVA) enrichment scores (ES) of cells, sorted by binding quintile for the TGF β_EMT DOWN gene set. **D, E**. GSVA enrichment scores (ES) of TGF β_EMT UP (D) and TGF β_EMT DOWN (E) gene sets for cell lines grouped by k-means computed clusters in Figure 1E.

**Supplementary Figure 3. The expression of many PG/GAG-related genes is correlated with TGF-β and canonical mesenchymal markers. A.** Heatmap of Pearson gene-gene correlation coefficients for GAG-related genes, cell surface proteoglycans, TGF-β isoforms and canonical markers of the mesenchymal phenotype; colored by gene function. **B.** Expression of select genes from A, within cell lines grouped by k-means computed EMT clusters from Fig 1E.

**Supp Figure 4**

**A..**

**B..**

**C..**

**D..**

**Supplementary Figure 4. Inhibition of TGF-β signaling is the major upstream regulator of the gene expression differences observed in the bel-sar resistant HeLa clones. A.** Genes (all capitalized) or gene complexes (mix of capital and lower-case characters) calculated by Ingenuity Pathway Analysis (IPA) as upstream regulators for the differential gene expression (DGE) signatures present in the bel-sar resistant cell lines. **B.** IPA regulation map of predicted downstream effectors of TGF-β1 signaling, overlaid with values from DGE comparing RxHela and bel-sar resistant HeLa cells. **C.** Quantification of western blot EMT markers relative to Parental HeLa. **D.** Proliferation of cell lines relative to Parental HeLa. Cells plated at 2000 cells per well, incubated for 48 hr and proliferation measured using XTT assay (R & D Systems).

**Supplementary Figure 5. Bel-sar selected HeLa clones have reduced expression of HS/CS. A-C.** Geometric mean fluorescence intensity (GMFI) of cells bound with bel-sar (A) or stained with anti-HS (B) or anti-CS antibodies (C). **D, E**. HPLC-SAX analysis of digested parental, RxHela, and bel-sar selected cell lines, for heparan sulfate (D) and chondroitin sulfate (E) disaccharides. Legend annotations: A: N-acetylglucosamine; a: N-acetylgalactosamine; D: Δ^4,5^- unsaturated uronic acid; S: N-sulfoglucosamine; adjacent number is the position of the sulfated carbon. **F.** GMFI-measured binding of bel-sar to cell lines in varying concentrations of FBS.

**Supplementary Figure 6. TGF-β signaling influences the expression of PG/GAG genes and extracellular PGs. A.** Dose response curve of GMFI calculated binding and bel-sar concentration, used for calculation of AUC in Figure 6A. **B.** Western blot of SMAD2 phosphorylation across HeLa cell lines untreated or treated with 10ng/mL TGF-β1 for 1 hr. **C**. Principal component analysis of RNA-seq gene expression from treatments in A (SB431542, SB). **D.** Gene expression changes of PG/GAG-related genes and cell surface proteoglycans within RxHeLa cells either untreated or after treatment with either 4µM SB431542 or 10ng/mL TGF-β1 for 72 hours. Expression depicted as log2 of the fold change compared to the untreated condition. SB431542 treatment expression levels indicated by vertical line; TGF-β1 treatment expression levels indicated by arrowhead. **E.** Gene expression changes of select extracellular PG genes within bel-sar selected cell lines upon treatment with 10ng/mL TGF-β1. Expression depicted as log2 of the fold change between cell clone and untreated RxHeLas.

**Supplementary Table 1.** Cell lines used in the study.

**Supplementary Table 2.** PG/GAG gene list with annotations
